# Use of genomics to design a diagnostic assay to discriminate between *Streptococcus pneumoniae* and *Streptococcus pseudopneumoniae*

**DOI:** 10.1099/mgen.0.000175

**Published:** 2018-04-09

**Authors:** Matthew A. Croxen, Tracy D. Lee, Robert Azana, Linda M. Hoang

**Affiliations:** ^1^​ BC Centre for Disease Control Public Health Laboratory, Vancouver, Canada; ^2^​ Provincial Laboratory for Public Health (ProvLab), University of Alberta Hospital, Edmonton, Alberta, Canada; ^3^​ Department of Laboratory Medicine and Pathology, University of Alberta, Edmonton, Canada; ^4^​ Department of Pathology and Laboratory Medicine, The University of British Columbia, Vancouver, Canada; ^‡^​ Present address: Provincial Laboratory for Public Health, Edmonton, Canada.; ^§^​ Present address: Department of Laboratory Medicine & Pathology, University of Alberta, Edmonton, Canada.

**Keywords:** *Streptococcus pseudopneumoniae*, genomics, real-time PCR

## Abstract

Distinuishing the species of mitis group streptococci is challenging due to ambiguous phenotypic characteristics and high degree of genetic similarity. This has been particularly true for resolving atypical *Streptococcus pneumoniae* and *Streptococcus pseudopneumoniae*. We used phylogenetic clustering to demonstrate specific and separate clades for both *S. pneumoniae* and *S. pseudopneumoniae* genomes. The genomes that clustered within these defined clades were used to extract species-specific genes from the pan-genome. The *S. pneumoniae* marker was detected in 8027 out of 8051 (>99.7 %) *S. pneumoniae* genomes. The *S. pseudopneumoniae* marker was specific for all genomes that clustered in the *S. pseudopneumoniae* clade, including unresolved species of the genus *Streptococcus* sequenced by the BC Centre for Disease Control Public Health Laboratory that previously could not be distinguished by other methods. Other than the presence of the *S. pseudopneumoniae* marker in six of 8051 (<0.08 %) *S. pneumoniae* genomes, both the *S. pneumoniae* and *S. pseudopneumoniae* markers showed little to no detectable cross-reactivity to the genomes of any other species of the genus *Streptococcus* or to a panel of over 46 000 genomes from viral, fungal, bacterial pathogens and microbiota commonly found in the respiratory tract. A real-time PCR assay was designed targeting these two markers. Genomics provides a useful technique for PCR assay design and development.

## Data Summary

1. All sequencing data done by the BC Centre for Disease Control Public Health Laboratory has been deposited to NCBI’s short read archive (SRA) under BioProject: PRJNA428833

2. All supplementary data can be found in Table S1 (available in the online version of this article)

**Impact Statement**

Mitis group streptococci are often difficult to distinguish with traditional biochemical assays. In particular, *Streptococcus pseudopneumoniae* is often hard to differentiate from atypical *Streptococcus pneumoniae*. Due to this, our understanding of the epidemiology and clinical significance of *S. pseudopneumoniae* has been limited. We sought to develop a suitable marker for distinguishing *S. pseudopneumoniae* from other species of the genus *Streptococcus* by using publicly available genomes along with phylogenetic support. This work should have broad interest for those studying species of the genus *Streptococcus*, in addition to being an example of how genomics can support the development of diagnostic assays.

## Introduction

*Streptococcus pseudopneumoniae* was first reported in 2004 by Arbique *et al*., and was described as an acapsular, bile-insoluble and optochin-resistant bacteria when grown in CO_2_. It is also a member of the mitis group streptococci [1]. Members of the mitis group streptococci can be difficult to identify to the species level and often lack genetic markers for reliable discrimination. For example, Arbique *et al*. showed that common pneumococcal targets, such as pneumolysin (*ply*) and autolysin (*lytA*) could be detected in a few *Streptococcus mitis* and the majority of *S. pseudopneumoniae* [1]. Studies by Kawamura *et al.* [2] and Wessels *et al.* [3] further illustrate the challenges with using 16S rRNA gene sequencing [2], biochemical, MALDI–TOF MS and molecular assays [3] in discriminating between members of the mitis group streptococci.

Given the challenges in resolving mitis group streptococci, the epidemiology and clinical significance of *S. pseudopneumoniae* is unclear. Pathogenicity of *S. pseudopneumoniae* has been shown in a murine model [4], while in humans, it has been associated with chronic obstructive pulmonary disease (COPD) [5]; others did not make the same observation [6]. A common feature of *S. pseudopneumoniae* appears to be the prevalence of erythromycin, tetracycline and penicillin resistance [5–8]. The paucity of studies on *S. pseudopneumoniae* have been undoubtedly hampered by challenges in distinguishing *S. pseudopneumoniae* from atypical *Streptococcus pneumoniae*.

*S. pseudopneumoniae* is genetically similar to *S. pneumoniae* according to the results of a genomic comparison study done by Shahinas *et al*. [9], which documented various shared and unique features between *S. pneumoniae*, *S. pseudopneumoniae* and *S. mitis*. Multilocus sequence analysis (MLSA) has been successful in a number of studies in discriminating mitis group streptococci [8, 10, 11]. In the same spirit as MLSA discriminates species of the genus *Streptococcus*, we used phylogenetic inference to look at the population structure of mitis group streptococci, irrespective of taxonomic classification in NCBI. Ultimately, we used this clustering information to inform marker discovery that was used to develop a real-time PCR assay that discriminates between *S. pneumoniae* and *S. pseudopneumoniae*.

## Methods

### *Streptococcus* growth conditions, and isolate selection for sequencing

Members of the genus *Streptococcus* referred to the British Columbia Centre for Disease Control Public Health Laboratory (BCCDC PHL) were selected for study. These isolates, though identified as belonging to the mitis group streptococci by partial 16S rRNA gene sequencing, could not be classified definitively as *S. pneumoniae*, *S. mitis* or *S. pseudopnuemoniae*. All isolates of members of the genus *Streptococcus* were grown on 5 % Columbia Sheep Blood Agar (Oxoid) at 37 °C in a CO_2_ incubator for 18–24 h. Fifty strains of members of the genus *Streptococcus* isolated from various sample types were selected; three of these isolates were identified as *S. pneumoniae*, one as *Streptococcus gordonii* and one as *Streptococcus australis*. The remaining 44 (plus one repeated sample) isolates belong to the mitis group streptococci, but after 16S rRNA sequencing had uncertain laboratory identification beyond the viridans grouping. ATCC strains *S. mitis* (ATCC 49456^T^), *Streptococcus oralis* (ATCC 9811), *S. pneumoniae* (ATCC 49619) and *S. pseudopneumoniae* (ATCC BAA-960^T^) were included as controls for the real-time PCR.

### Genome sequencing

Nucleic acids were extracted from the isolates of members of the genus *Streptoccocus* using a DNeasy Blood and Tissue Kit (QIAgen) or a MaxMAX DNA Multi-Sample Ultra Kit (ThermoFisher). The extracted DNA was made into Illumina-compatible libraries using either a Nextera XT (Illumina), TruSeq Nano DNA Library Prep Kit for NeoPrep (Illumina) or a NxSeq AmpFREE Low DNA Library Kit (Lucigen). Libraries made with the NxSeq AmpFree DNA Library Kit were quantified using the NEBNext Library Quant Kit for Illumina (New England BioLabs). All libraries were sequenced on an Illumina MiSeq using a 500-cycle MiSeq V2 kit (Illumina). Quality of the raw sequencing reads was assessed using FastQC v0.11.5 (www.bioinformatics.babraham.ac.uk/projects/fastqc/) and MultiQC 1.2 [12]. One isolate, BCCDCPHL-Ssp027, failed to sequence and was not further analyzed. All raw sequence data is available from the BCCDC PHL Genomic Data Bank (BioProject: PRJNA379148), specifically this study under BioProject: PRJNA428833.

### Genome assembly

Raw Illumina reads were adapter and quality trimmed with Trimmomatic v0.36 [13], using the adapter sequences packaged with the A5-miseq assembly pipeline [14]. The resulting trimmed reads were assembled with the Unicycler 0.4.1 [15] assembly pipeline with the - -no_pilon option, using SPAdes v3.11.0 [16] as the assembler for the trimmed Illumina reads.

### Public genome download

All available genomes of members of the genus *Streptococcus* from RefSeq release 84 were downloaded using ncbi-genome-download 0.2.5 (github.com/kblin/ncbi-genome-download) (*n*=11 455). In addition, we downloaded *S. pseudopneumoiae* sequence data from BioProjects PRJEB20507, PRJEB4909, PRJEB2340 and PRJNA225866, and assembled the genomes, where appropriate, as described above (*n*=16). In total, 52 *S. pseudopneumoniae* genomes (including one labelled *S. mitis*) were gathered (Table S1). Non-streptococci genomes that were used to assess the analytical specificity (exclusivity) were also downloaded with ncbi-genome-download (*n*=46 727), and included microbiota found in respiratory samples [17].

### Phylogenetic inference of *Streptoccocus* spp

Genomes were used to reconstruct a phylogenetic tree using PhyloSift v1.0.1 [18], which places genomes phylogenetically using 37 reference markers that are found in single copies and are nearly universal. The alignment of these phylogenetic markers (21 327 nucleotide positions) were used to infer a maximum-likelihood tree using a generalized time-reversible (GTR) +GAMMA evolutionary model, and 100 bootstraps with RAxML 8.2.8 [19]. Trees were pruned and labels edited using newick_utils (github.com/tjunier/newick_utils). Phylogenetic trees were visualized using plotTree.py (github.com/katholt/plotTree), a wrapper script for Environment for Tree Exploration (ETE) [20].

### Marker discovery

The pan-genome of the 34 complete RefSeq *S. pneumoniae* genomes and 27 S*. pseudopneumoniae* genomes (based on their phylogenetic placement) were generated using large-scale blast score ratio (LS-BSR) v1.011 analysis [21], predicting genes with Prodigal v2.6.3 [22] and clustering using VSEARCH v2.5.0 [23]. The LS-BSR accessory script (compare_BSR.py) was used on the resulting LS-BSR gene matrix to compare and extract genes that were unique to all 34 *S. pneumoniae* or 27* S. pseudopneumoniae*. Candidate markers for either *S. pneumoniae* or *S. pseudopneumoniae* were selected based on having a sequence length longer than 500 nucleotides and over 99 % identity (number of identical nucleotides of query divided by subject length) when aligned back to all originating genomes using blastn v2.6.0+ [24]. Other bioinformatics software, such as bioawk (github.com/lh3/bioawk), and seqtk (github.com/lh3/seqtk) were used to filter and manage the sequence data. Candidate markers were annotated using prokka v1.12 [25].

### *In silico* Multilocus Sequencing Typing (MLST)

Sequence types were assigned to genomes of interest using mlst 2.10-dev (github.com/tseemann/mlst) with the *S. pneumoniae* mlst database downloaded on October 26, 2017.

### *In silico* pneumococcal capsule typing

Assembled genomes were used to simulate Illumina sequencing data at a sequencing depth of 150× with wgsim 0.3.2 (https://github.com/lh3/wgsim). These data were used with Pneumococcal Capsule Typing (PneumoCaT 1.0) pipeline [26] to predict pneumococcal serotypes from Illumina sequence data.

### Taxonomic classification of discrepant isolates

Discrepant classifications were assessed by simulating the Illumina sequences using the assembled genome in question with wgsim 0.3.2 at a sequencing depth of 150×. The simulated reads were classified using Kraken version 1.0 [27] using the minikraken_20171019_8 GB database, and the most likely taxonomy was based on the classification with the largest number of reads assigned to it.

### Real-time PCR assay

A TaqMan assay was developed for the *S. pneumoniae* marker (SPN0001) and *S. pseudopneumoniae* marker (SPS0002). Primers and probes were designed using Geneious 9.0.4 (www.geneious.com, [28]) (Table 1), IDT OligoAnalyzer 3.1 was used to assess primer interactions, and Thermofisher Primer Express 3.0.1 to predict primer and probe melting temperatures. Real-time PCR reactions were performed on an ABI 7500 with recommended Fast thermal-cycling conditions in a 20 µl final volume using TaqMan Fast Advanced Master Mix (Life Technologies), PCR-grade water and primers and probes in a 20× mix. The 20× multiplex mix consists of each primer and probe resuspended in IDTE (pH 8.0) at a final concentration of 200 nM each for SPN-F and SPN-R, 100 nM for SPN-P, 500 nM each for SPS-F and SPS-R and 250 nM for SPS-P oligonucleotides.

**Table 1.** Oligonucleotides and probes for amplification of SPN0001 and SPS0002

Target	Organism	Name	Sequence	Probe
SPN0001	*S. pneumoniae*	SPN-F	AATATCTGAAGATGCTCATTCTACAATT	
		SPN-R	ATAAGGTTTACCGTCAATAATACGCAG	
		SPN-P	AACTACAGGTCGCTTTGCAGAGTCCAGTTT	6FAM/ZEN
SPS0002	*S. pseudopneumoniae*	SPS2-F	GTTCGGACTGGAGAGGAAGC	
		SPS2-R	AAGCTACGAATCTTGTCAATAATGTCTT	
		SPS2-P	ACAGATCATTTCGCAATTT	VIC MGB

*Streptococcus* lysates were prepared by either re-suspending a half loop (0.01 ml) of bacterial growth from isolated colonies into 1 ml of PCR-grade water in a micro-centrifuge tube and heating in a dry bath at 100 °C for 8 min, or using Instagene following the manufacture’s protocol. A 2 µl aliquot of sample lysate was used in each real-time PCR reaction.

## Results and discussion

**Fig. 1.**
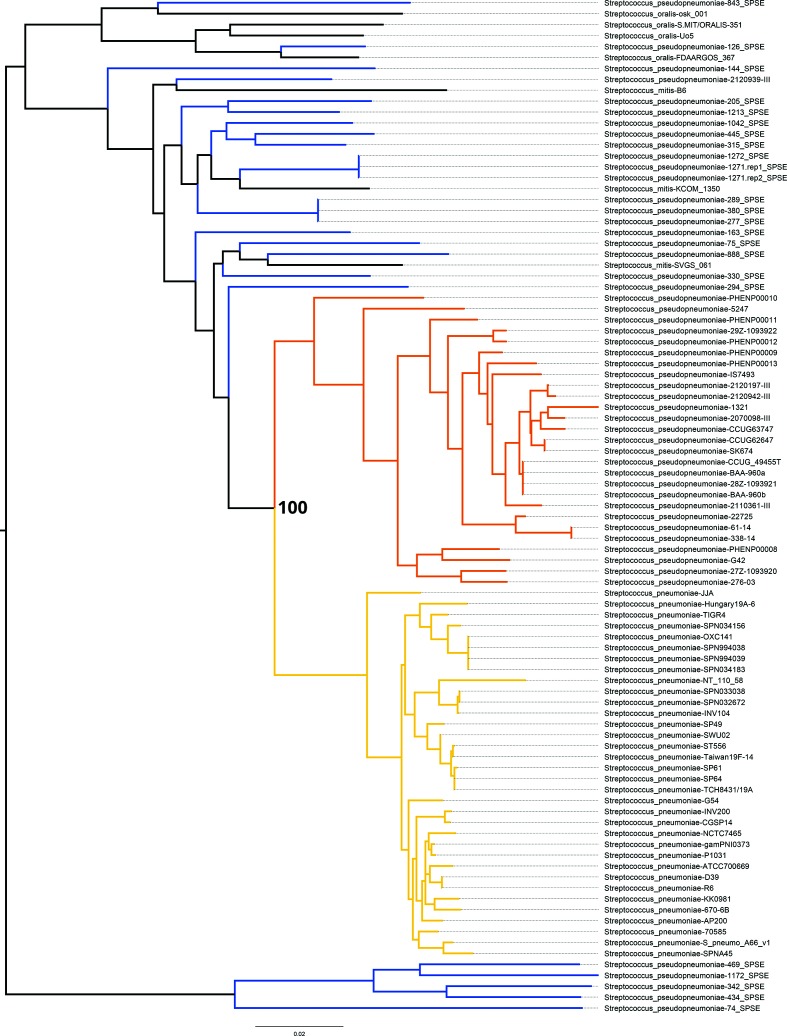
Phylogenetic tree of selected species of the genus *Streptococcus*. Phylosift was used to place 34 *S. pneumoniae* (gold branches), 52 *S. pseudopneumoniae*, three *S. mitis* and four *S. oralis* based on NCBI taxonomy (RefSeq release 84). Note that one *S. mitis* strain (1042_SPSE) was included as a *S. pseudopneumoniae* due to information in supplemental data from [29]. The *S. pneumonaie* cluster is shown with gold branches, while the major *S. pseudopneumoniae* cluster (including two *S. pseudopneumoniae* ATCC BAA-960^T^ genomes) is shown with orange branches. *S. pseudopneumoniae* that fall outside of the orange *S. pseudopneumoniae* clade are denoted by blue branches and were ultimately excluded from the marker discovery process. Bootstrap support values are indicated in black bold type at the node that separates the *S. pneumoniae* and *S. pseudopneumoniae* clades.

We set out to look for species-specific markers that would unambiguously distinguish *S. pseudopneumoniae* from *S. pneumoniae* and other members of the mitis group streptococci. Given the challenges related to accurately identifying *S. pseudopneumoniae*, we elected to first generate a phylogenetic tree of species of the genus *Streptoccocus* downloaded from RefSeq complete, including 34 *S. pneumoniae*, four *S. oralis* and three *S. mitis*, as well as 36 *S. pseudopneumoniae* and 16 more from various BioProjects (Table S1). Fig. 1 illustrates that 25 *S. pseudopneumoniae* cluster (blue branches) among *S. mitis,* and *S. oralis*. Of the 52 *S. pseudopneumoniae* isolates 27 clustered together on the tree in a clade (orange branches) near the *S. pneumoniae* clade (gold branches), including two *S. pseudopneumoniae* ATCC BAA-960^T^ genomes that had been sequenced by different groups. The majority (24 out of 25) of the *S. pseudopneumoniae* that were not part of the large (orange) *S. pseudopnuemoniae* clade were isolated and sequenced during a study in one intensive care unit population [29]. In that study, the original laboratory identification of these *S. pseudopneumoniae* were mostly ‘Strep Viridans’, *Neisseria*, *Enterococcus fecaelis* or *Staphylococcus aureus*, while the authors used the average nucleotide identity (ANI) to find the best match of these 24 genomes to *S. pseudopneumoniae* IS7493 in the NCBI database. The taxonomy that was applied to these genomes and uploaded to NCBI was based on the ANI, which ranged from 0.82 and 0.93. It has been suggested that an ANI of at least 0.95 is needed for classification of isolates as members of the same species [30–32]. Given that the *S. pseudopneumoniae* classification from the Roach *et al*. study [29] is not strongly supported, we decided to use the *S. pseudopneumoniae* genomes that clustered within that defined clade (Fig. 1; orange clade). *S. pseudopneumoniae* 2120939-III (BioProject: PRJEB4909), also did not cluster within the defined clade and was excluded from the marker discovery process (Fig. 1, and Table S1).

### *S. pneumoniae* and *S. pseudopneumoniae* have specific markers

We next wanted to look for *S. pneumoniae* and *S. pseudopneumoniae* species-specific markers. To accomplish this, we took all genomes complete RefSeq *S. pneumoniae* (*n*=34) and publicly available *S. pseudopneumoniae* genomes that clustered within the *S. pseudopneumoniae* phylogenetic clade (Fig. 1; *n*=27) and identified the pan-genome using LS-BSR [21]. After filtering, 13 candidate *S. pneumoniae* and four *S. pseudopneumoniae* genes that were greater than 500 nucleotides in length were identified. These had at least a 99 % identical match across their intended targets (Table S1). We decided to further investigate a single candidate marker from both *S. pneumoniae* (centroid_2470; 729 nt; GtnR-family transcriptional regulator; SPN0001) and *S. pseudopneumoniae* (centroid_2440; 735 nt; *kdpDE—*an osmosensitive potassium channel histidine kinase/response regulator; SPS0002). Analytical specificity (inclusivity) was assessed by looking for blast hits for each marker against all species of the genus *Streptococcus* (*n*=11 455) and the non-RefSeq *S. pseudopneumoniae* (*n*=16), to look for any potential-cross reactivity within the genus.

The *S. pneumoniae* marker was found in 8019 out of 8066 (99.41 %) of the *S. pneumoniae* genomes in RefSeq, it was not found in any non-pneumococcal genomes. The criteria for a genome containing a marker required at least 99 % nucleotide identity across the length of the SPN0001 target. If we looked at the raw blastn output before applying the strict 99 % identity across the entire gene, there were 11 out of 47 *S. pneumoniae* genomes that had blastn matches with fewer identical nucleotides. When we queried these 11  *S. pneumoniae* genomes with the 154 base pair (bp) real-time PCR marker sequence (see below), eight of them matched the SPN0001 target with at least 99 % nucleotide coverage (Table S1; spn0001_discrepant). The remaining 3 *S. pneumoniae* isolates had short blastn matches, which may be reflective of misassemblies or inadequate genome sequencing coverage prior to assembly. On the basis of the results of the the *in silico* analysis using the SPN0001 PCR target sequence, the adjusted specificity of SPN0001 would improve to 8027 out of 8066 (99.52 %).

The *S. pseudopneumoniae* marker was found in all 27 *S. pseudopneumoniae* used to discern the marker, as expected, and did not have any matches in the 25 so-called *S. pseudopneumoniae* that did not cluster within the major *S. pseudopneumoniae* clade (Fig. 1). There were, however, 20 non-*pseudopneumoniae* matches: two *Streptococcus canis* and four *Streptococcus pseudoporcinus* that shared approximately 80 % identical nucleotide sequence to SPS0002, and 14 *S. pneumoniae* genomes (14 out of 8066; 0.173 %). We looked at the MLST of the 14 *S. pneumoniae* and five of them represent ST5107 (non-typeable according to PneumoCaT [26]), isolated from Thailand during a study by Chewapreecha *et al*. [33], and one was a ST2971 from China (also non-typeable). The remaining eight genomes belong to various unknown sequence types and were all non-typeable except for one serotype 37 (Table S1; sps0002_discrepant), a serotype that has been described in non-pneumococcal streptococci [34].

We also looked at the exclusivity of these two markers by assessing any blast hits to known viral, fungal and bacterial (microbiota and pathogens) genomes associated with sputum and nasopharyngeal samples (Table S1; exclusivity). Of the 46 727 genomes queried, only the *S. pseudopneumoniae* marker, SPS0002, matched 38 identical nucleotides (38 out of 735 nt; 5 %) in four species of the genus *Enterococcus* (Table S1; sps0002_discrepants). On the basis of analytical specificity inclusivity and exclusivity results, both the *S. pneumoniae* SPN0001 and *S. pseudopneumoniae* SPS0002 markers have high specificity to their respective species and were considered useful targets for a real-time PCR assay.

### Presence/absence of *S. pneumoniae* (SPN0001) and *S. pseudopneumoniae* (SPS0002) PCR marker is concordant with phylogenetic placement of clinical isolates of species of the genus *Streptococcus*

The initial impetus for this study was to develop molecular markers that would distinguish *S. pneumoniae* from *S. pseudopneumoniae*. Since the SPN0001 and SPS0002 markers were used to look for presence and absence of all RefSeq streptococci genomes, we added discrepant results and more genomes to the reference phylogenetic tree (Fig. 1) to further understand the relationship of these markers to the clustering of the genomes. We included all *S. pneumoniae* isolates that lacked the SPN0001 PCR target (154 nucleotide sequence; *n*=39; originally 47, but excluding the eight *S. pneumoniae* that had truncated SPN0001, but that contained the PCR marker as described above), as well as all *S. pneumoniae* isolates that contained the SPS0002 PCR target (119 nucleotide sequence; *n*=14). Two *S. mitis*, one *S. oralis* and five *S. infantis* genomes randomly picked from RefSeq were added to populate the tree such that each species was represented by five members of these mitis group streptococci. Finally, BCCDC PHL sequenced streptococci isolates were also included on the tree, but are described below.

**Fig. 2.**
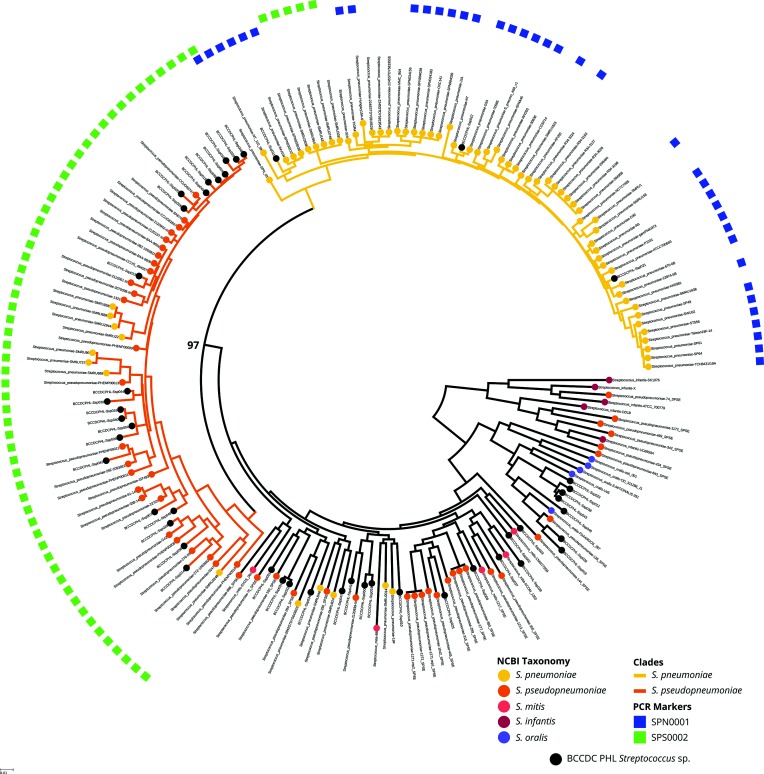
Phylogenetic tree of members of the genus *Streptococcus* and the presence/absence of the SPN0001 and SPS0002 PCR marker. A phylogenetic tree was reconstructed to show the relationship of the SPN0001 *S. pneumoniae* (blue squares) and SPS0002 *S. pseudopneumoniae* (green squares) PCR markers and the placement of the genomes on the tree. Note the *S. gordonii*, and S. *australis* isolates were excluded from this tree. *S. pneumoniae* (gold nodes); *S. pseudopneumoniae* (orange nodes); *S. mitis* (coral nodes); *S. oralis* (mauve nodes); *S. infantis* (magenta nodes); and BC *Streptococcus* sp. (black nodes). Bootstrap support value is indicated in black bold type at the node that separates the *S. pneumoniae* and *S. pseudopneumoniae* clades. All SPN0001-positive genomes clustered in the *S. pneumoniae* clade (yellow branches) while almost all SPS0002-positive genomes clustered in the *S. pseudopneumoniae* clade (orange branches). The three BC *S. pneumoniae* clinical isolates clustered with the other *S. pneumoniae,* while 22 BC SPS0002-positive *Streptoccocus* sp. clustered with the *S. pseudopneumoniae* clade. This tree also shows all discrepant results (excluding non-*Streptococcus* organisms and *S. salivarius*), such as SPN0001-negative *S. pneumoniae* and SPS0002-positive *S. pneumoniae*. Note that only RefSeq complete *S. pneumoniae* and discrepant *S. pneumoniae* genomes are shown as a representation of all 8051 *S. pneumoniae* genomes in RefSeq. Overall, 8027 out of 8051 *S. pneumoniae* were SPN0001-positive.

The first tree that was generated had two isolates that were distantly related to the other isolates on the tree. We were suspicious of the classification of these organisms, which were labelled *S. pneumoniae* in the NCBI records. We simulated raw reads from both of these two genomes and used Kraken to classify the simulated reads. One isolate was classified as *Streptococcus salivarius*, while the other was classified as a *Staphylococcus* species. Further evidence that these were not *S. pneumoniae* genomes came from the *in silico* MLST results; the *S. salivarius* genome had no matches to any *S. pneumoniae* MLST markers, while the *Staphylococcus* sp. genome best matched MLST markers from *Stapyhlococcus hominis* (Table S1). We pruned these two genomes from the phylogenetic tree and regenerated the tree, decorated with blast results for both the SPN0001 and SPS0002 real-time PCR sequence target (Fig. 2).

The presence and absence of the *S. pneumoniae* (SPN0001) and *S. pseudopneumoniae* (SPS0002) PCR marker sequences correlated almost exclusively with the genomes that clustered in the *S. pneumoniae* (Fig. 2; gold clade) and *S. pseudopneumoniae* (Fig. 2; orange clade) clades. One exception was a *S. pneumoniae* genome (ST2971) that was both SPN0001- and SPS0002-positive, but clustered with the *S. pneumoniae* clade.

We noted that many of the discrepant genomes identified above may be due to incorrect classification in the NCBI RefSeq record, as they clustered according to the presence of either the SPN0001 or SPS0001 PCR markers. Many of the taxonomic classification associated with genomes in NCBI RefSeq originate from the submitting laboratory, and the two genomes (*S. salivarius* and the species of the genus *Staphylococcus*) that were pruned from the final tree (Fig. 2) made us suspicious of some of the other discrepant genomes. To provide a reference method to classify these genomes, we used the top match from Kraken classification to support or refute the classification provided by NCBI. For *S. pneumoniae* that were SPN0001-negative, 15 genomes clustered outside of the *S. pneumoniae* clade (Fig. 2; gold clade): two were pruned from Fig. 2 (*S. salivarius* and the member of the genus *Staphylococcus*), five were classified as *S. mitis* and eight were classified as *S. pseudopneumoniae* (Table S1). The eight genomes that were classified *as S. pseudopneumoniae* clustered with the *S. pseudopneumoniae* clade (Fig. 2; orange clade) and were SPS0002-positive. The remaining 24 discrepant *S. pneumoniae* genomes clustered with the *S. pneumoniae* clade, but were SPN0001-negative. Those 24 *S. pneumoniae* consisted of 11 different MLST sequences types, and one unknown sequence type among these 24 *S. pneumoniae* genomes (Table S1). Notably, ST425 (*n*=6), ST5107 (*n*=5), and ST2705 (*n*=3) are present multiple times. In terms of serotype, 9 out of 24 genomes were predicted to be non-typeable whereas the rest were assigned a predicted serotype of 19F (*n*=7), 33F (*n*=3), 3, 06E, 14, 23F and 32F (Table S1; spn0001_discrepants). The five *S. pneumoniae* ST5107 isolates were SPN0002-positive, and along with the *S. pneumoniae* ST2971 (SPN0001- and SPS0002-positive) are the only instances of the SPS0002 PCR marker having a match outside of genomes in the *S. pseudopneumoniae* clade. This is possibly due to recombination, common in *S. pneumoniae* and particularly in acapsular lineages [33]. With the support of the Kraken classification and the tree placement for 15 discrepant *S. pneumoniae* genomes, we readjusted the SPN0001 specificity to 8027 out of 8051 (99.70 %). Likewise, the *S. pseudopneumoniae* SPS0002 marker specificity to *S. pneumoniae* was adjusted based on 8 out of 14 *S. pneumoniae* genomes probably being *S. pseudopneumoniae* (they cluster within the *S. pseudopneumoniae* clade and were SPS0002-positive), and SPS0002 marker was detected in 6 out of 8051 (0.074 %) of *S. pneumoniae* genomes.

Over a three-year period, the BCCDC PHL collected isolates of members of the genus *Streptococcus* that could not be classified to species by 16S rRNA gene sequencing. We took this collection of unknown isolates of members of the genus *Streptococcus* and sequenced their genomes to see where they clustered phylogenetically, and if that clustering was supported by the expected matches to the SPN0001 and SPS0002 PCR markers. We grew 44 ambiguous isolates of members of the genus *Streptococcus* from April 10 2014 to June 1, 2017 for genome sequencing, as well three laboratory-confirmed *S. pneumoniae* isolates, one *S. gordonii* isolate and one *S. australis* isolate. All isolates, with the omission of BCCDCPHL-Ssp027 (failed sequencing), *S. gordonii*, and *S. australis*, were added to the reference phylogenetic tree seen in Fig. 1 to generate the final tree (Fig. 2). The three clinically identified *S. pneumoniae* clustered with the other *S. pneumoniae* genomes and were positive for the SPN0001 PCR marker. The remaining 45 (44 plus one repeat) genomes of members of the genus *Streptococcus* clustered throughout the phylogenetic tree, however, all SPS0002-positive genomes (*n*=22) clustered with the *S. pseudopneumoniae* clade, indicating that they are probably isolates of *S. pseudopneumoniae*. Together, the SPS0002 PCR marker was detected in 57 out of 57 (27 RefSeq genomes, eight Kraken genomes classified as *S. pseudopneumoniae* and 22 BC sequenced genomes) *Streptococcus* genomes when they clustered within the *S. pseudopneumoniae* clade. Finally, we looked at the presence/absence, of the *S. pneumoniae* R6 *lytA* gene (Accession number: NC_003098.1; locus_tag:spr1754) among the isolates included on the tree in Fig. 2. This *lytA* gene was detected in all genomes from both the *S. pneumoniae* and *S. pseudopneumoniae* clades at over 98 % and approximately 82 % nucleotide identity, respectively (Table S1). However, *lytA* was also detected in 20 SPN0001- and SPS0002-negative genomes, such as *S. mitis* B6, at approximately 82 % nucleotide identity. These data further support the usefulness of SPS0002 for distinguishing *S. pseudopneumoniae* from *S. pneumoniae*.

### Real-time PCR assay results agree with phylogenetic placement of *S. pneumoniae* and *S. pseudopneumoniae* isolates

Given the *in silico* specificity of the SPN0001 PCR marker to *S. pneumoniae* and specificity of the SPS0002 PCR marker to *S. pseudopneumoniae*, these sequences were designed as a real-time PCR assay (Table 1), which can be run as a singleplex or a duplex. We had three different panels: (1) A well-characterized panel (*n*=36), consisting of *S. pneumoniae* serotyped at the National Microbiology Laboratory (Winnipeg, Manitoba, Canada), ATCC and DSMZ isolates; (2) a clinical panel made up of 103 clinical isolates of members of the genus *Streptococcus* identified by the BCCDC PHL and; (3) the 49 (plus one repeat) isolates of members of the genus *Streptococcus* that had been sequenced on the Illumina MiSeq (Table S1).

SPN0001 was detected in all known isolates of *S. pneumoniae* in all three panels (29 out of 29) with 100 % accuracy and analytical specificity; no cross-reactivity was observed in the remaining 159 isolates of members of the genus *Streptococcus* (Table S1). SPS0002 could be detected in only *S. pseudopneumoniae* ATCC BAA-960^T^, as expected, from the well-characterized panel. In the clinical panel, ten isolates of the *S. mitis* group were positive for SPS0002, indicating that they were probably *S. pseudopneumoniae*. However, because of the lack of a reference assay that could reliably confirm the species classification of mitis group streptococci, the detection of the SPS0002 marker in the genome sequencing panel and where the SPS0002-positive isolates clustered on the tree was important. The real-time PCR results confirmed the clustering of the sequenced isolates of members of the genus *Streptococcus* on the phylogenetic tree in Fig. 2; all genomes of members of the genus *Streptococcus* that clustered within the *S. pseudopneumoniae* clade (Fig. 2; orange clade) were SPS0002-positive, while any members of the genus *Streptococcus* not clustering within the *S. pseudopneumoniae* cluster were PCR negative for SPS0002. These data support the hypothesis that the SPN0001 and SPS0002 markers identified using comparative genomics are suitable markers for distinguishing *S. pneumoniae* and *S. pseudopneumoniae* from other mitis group streptococci.

In this study we used the power of genomics to identify molecular specific markers capable of reliably differentiating *S. pneumoniae* and *S. pseudopneumoniae*. These markers were used as the basis for development of a real-time PCR assay, providing the clinical, microbiology and epidemiological communities a robust tool for reliable differentiation of *S. pneumoniae* and *S. pseudopneumoniae*. Given the abundance of misidentification of mitis group streptococci in NCBI RefSeq, phylogenetic inference was helpful in separating species to give us confidence in the dataset that we used to capture specific markers from the pan-genome. The phylogenetic inference was also helpful for looking at discrepant results as we were testing our markers *in silico*. For example, during our *in silico* analysis of the *S. pseudopneumoniae* marker, 14 *S. pneumoniae* matches (out of 8051) were found. Clustering of these 14 discrepant *S. pneumoniae* genomes placed eight of them in the *S. pseudopneumoniae* clade, and these eight genomes were also negative for the *S. pneumoniae* marker. This highlights the importance of alternate methods to investigate discrepant genomes from public databases, such as NCBI RefSeq. Database issues aside, this approach has broad applications for other diagnostics, including targeted assay design for outbreaks or surveillance, similar to that described by Bowers *et al*. [35].

## Data bibliography

1. Croxen MA, Lee TD, Azana R, Hoang LM. BioProject:PRJNA428833 (2018).
2. Croxen MA, Lee TD, Azana R, Hoang LM. Supplemental Table 1 (2018).
3. NCBI RefSeq release 84. www.ncbi.nlm.nih.gov/refseq/ (2017).
4. University of Oxford PubMLST. www.pubmlst.org (October 26, 2017).
5. Roach DJ, Burton JN, Lee C, Stackhouse B, Butler-Wu SM *et al*. https://doi.org/10.1371/journal.pgen.1005413.s012 (2015).
6. Public Health England BioProject: PRJEB20507 (2017).
7. Wellcome Trust Sanger Institute BioProject:PRJEB4909 (2014), used with permission.
8. Ikryannikova LN, Ischenko DS, Lominadze GG, Kanygina AV, Karpova IY *et al*. BioProject: PRJNA225866 (2013).
9. Wellcome Trust Sanger Institute Bioproject: PRJEB2340 (2013), used with permission.

## Supplementary Data

Supplementary File 1
